# Supplementary material for: Active site specificity profiling datasets of matrix metalloproteinases (MMPs) 1, 2, 3, 7, 8, 9, 12, 13 and 14
Source: Data Brief. 2016 Feb 22;7:299–310. doi: 10.1016/j.dib.2016.02.036 (PMC4777984; doi:10.1016/j.dib.2016.02.036)
Supplement: Supplementary file 10 — Supplementary material [file mmc10.zip › WebPICS_hMMP13_T_1%/P3prime.html]

 

PICS results


|  |  |
| --- | --- |
| **P3prime\_A**  29 in 143 sites   20.3 %    effects > 10 perc. pnts.   (vice-versa in brackets)  P2\_V: 11.6 (25.9)   P1\_A: 13.6 (19.7)   P1\_G: -11.3 (-12.6)   P2prime\_L: 10.9 (22.6) |  |
| **P3prime\_D**  14 in 143 sites   9.8 %    effects > 10 perc. pnts.   (vice-versa in brackets)  P1prime\_I: 16.7 (13.7)   P2prime\_K: 11.6 (11.6) |  |
| **P3prime\_G**  26 in 143 sites   18.2 %    effects > 10 perc. pnts.   (vice-versa in brackets)  P1\_G: 12.6 (12.6) |  |
| **P3prime\_H**  11 in 143 sites   7.7 %    effects > 10 perc. pnts.   (vice-versa in brackets)  P2\_F: 12.6 (17.3)   P2\_H: 15.4 (42.3)   P1\_P: 35.7 (28.0)   P1prime\_Q: 11.9 (14.5)   P2prime\_Q: 28.0 (25.6) |  |
| **P3prime\_N**  8 in 143 sites   5.6 %    effects > 10 perc. pnts.   (vice-versa in brackets)  P3\_A: 24.2 (10.2)   P2\_F: 19.4 (19.4)   P2prime\_I: 38.8 (19.4) |  |
| **P3prime\_S**  13 in 143 sites   9.1 %    effects > 10 perc. pnts.   (vice-versa in brackets)  P2\_Q: 17.5 (28.4)   P1prime\_M: 11.2 (24.2)   P2prime\_T: 25.2 (40.9)   P2prime\_W: 12.6 (40.9) |  |
| **P3prime\_T**  9 in 143 sites   6.3 %    effects > 10 perc. pnts.   (vice-versa in brackets)  P1\_D: 14.5 (11.9)   P1\_Q: 29.1 (43.7)   P1prime\_C: 16.6 (18.7) |  |
